# Lymphatic filariasis in Zambia: A scoping review protocol

**DOI:** 10.1371/journal.pone.0292237

**Published:** 2023-10-04

**Authors:** Hugh Shirley, Adrienne Orriols, Dylan Hogan, Kingford Chimfwembe, Alinaswe Balya, Kaala Sibbuku, Janelle Lardizabal, Sophie Tillotson, Philip Espinola Coombs, Richard Wamai

**Affiliations:** 1 Harvard Medical School, Boston, MA, United States of America; 2 African Center for Community Investment in Health, Nginyang, Baringo County, Kenya; 3 Climate, Obstetrics, Anesthesia, and Surgery Team (COAST), Program for Global Surgery and Social Change (PGSSC), Brigham and Women’s Hospital, Boston, MA, United States of America; 4 University of Florida College of Medicine, Gainesville, FL, United States of America; 5 College of Science, Northeastern University, Boston, MA, United States of America; 6 Ministry of Health, Zambia, Lusaka, Zambia; 7 Chreso University, Lusaka, Zambia; 8 Department of Behavioral Neuroscience, College of Science, Northeastern University, Boston, MA, United States of America; 9 Department of Research & Instruction, Northeastern University Library, Northeastern University, Boston, MA, United States of America; 10 Department of Cultures, Societies and Global Studies, College of Social Sciences and Humanities, Integrated Initiative for Global Health, Northeastern University, Boston, MA, United States of America; 11 Department of Global and Public Health, University of Nairobi, Nairobi, Kenya; 12 Nigerian Institute of Medical Research, Federal Ministry of Health, Lagos, Nigeria; Vector Control Research Centre, INDIA

## Abstract

**Background:**

Zambia is among the countries making major progress in limiting cases of the neglected tropical disease lymphatic filariasis on the path to reaching global elimination targets. For this trend to continue, it is essential for strategies and policies targeting the disease in Zambia to be based on the most recent and relevant literature. The scope of research on lymphatic filariasis in the Zambian context is currently poorly understood. Therefore, this study describes a scoping review protocol which will be used to analyze the body of literature on lymphatic filariasis in Zambia.

**Methods:**

The scoping review protocol was developed following the PRISMA reporting guidelines for Scoping Reviews (PRISMA-ScR) and the JBI Scoping Review Methodology Group’s guidance on conducting scoping reviews. In consultation with a research librarian, these guidelines will be applied to a literature search of articles from peer-reviewed journals, or government and international regulatory bodies using PubMed, Embase, Web of Science, Cochrane CENTRAL, WHO ICTRP, Pan African Clinical Trials Registry, and ClinicalTrials.gov. Each record will be screened at the abstract and full-text level by two independent reviewers, and results reported via summary statistics.

**Discussion:**

Understanding the current state of research on lymphatic filariasis in Zambia will identify major knowledge and intervention gaps in this context, and serve as a source of information for surrounding countries in the region. As the disease prevalence drops, efforts for elimination will require carefully targeted strategies which can be informed from the literature identified in this protocol.

## Introduction

### Background

Lymphatic filariasis (LF) is a mosquito-borne neglected tropical disease that is endemic in 72 countries, including Zambia [1–3]. In Africa, the disease is caused by *Wucheria bancrofti* filariae and is spread by *Anopheles* and *Culex* mosquitoes [4]. An estimated 51.4 million people were infected with LF in 2019, down from 199 million in 2000 [5]. The disease process involves chronic infection which leads to lymphatic dysfunction, resulting in disabling lymphedema and filarial hydrocele formation [2, 6, 7]. The resulting physical, psychological, and socioeconomic burden placed on patients and their families contributes to significant disability [7–9].

In 2000, the Global Programme to Eliminate LF (GPELF) was founded with the goal of global elimination by 2020; while this goal was not realized, great strides have been made in reducing its endemicity worldwide [5]. Mass drug administration (MDA) campaigns, involving annual population-level administration of pharmacologic prophylaxis, have served as the backbone for elimination efforts [5, 10–12]. To date, two countries in Africa, Malawi and Togo, have eliminated LF and four more are under post-MDA implementation surveillance prior to elimination validation [1, 13]. Besides MDAs for prophylaxis, treatment of active LF infection remains an important part of global LF work. Filarial hydroceles, which frequently occur in chronically infected males, require surgery for definitive repair [7, 8]. In 2021, 139,043 hydrocele patients were reported to WHO in Africa, however, this likely underestimates the true burden of this condition given remote patient populations and its associated stigma, among other challenges [1].

Zambia, too, has made progress towards eliminating LF through the implementation of MDAs and other public health campaigns [2]. Epidemiological surveys conducted between 2003–2011 estimated disease prevalence at 7.4% nationwide, with some regions, such as Western province, demonstrating prevalence as high as 53.9% [2, 3]. LF is endemic in 96 of 116 Zambian districts [14]. In 2015, over 10.7 million people received antihelminth medications as part of Zambia’s MDA, and 92.8% of endemic regions were effectively covered [15]. Year over year, the number of endemic regions has declined due to this successful campaign, and in 2021, 4.8 million people were treated, and 97.1% of endemic regions were effectively covered [1]. As programming to eliminate LF in Zambia continues, charting both the progress and research of the past few decades may provide insight into research gaps and new directions that may assist in achieving 100% MDA coverage and, ultimately, elimination.

Post-2020 and in the era of the COVID-19 pandemic, the NTD Roadmap 2021–2030 has redefined elimination goals for LF [11]. While elimination is still on the agenda, the roadmap established a more tempered goal of eliminating LF in 81% of endemic countries by 2030, rather than global elimination as previously established in 2000 [11]. Priorities include the development of more specific rapid diagnostic tests and improvements to post-MDA surveillance [11]. In this new NTD era for Zambia, understanding the current state of research on LF in the country is critical for optimizing future research goals to align with national and international LF goals and to adapt strategies to ongoing and new challenges to LF treatment and MDAs.

### Rationale

Given Zambia’s ongoing efforts to reduce the burden of LF across the country, an understanding of the state of research on LF in Zambia could be beneficial in both guiding future interventions and as a source of information for other countries in the region in which LF is endemic. Additionally, as the burden of lymphatic filariasis continues to decline in Zambia, new challenges may make additional progress incrementally more difficult. Defining the current state of research in Zambia, therefore, will provide insight into current gaps in understanding that may fuel future research direction. To date, no review of the state of LF research in Zambia has been performed. Here, we present a protocol for a scoping review being conducted on the state of LF research in Zambia to fill this gap.

### Objective

The study objective is to define the scope of peer-reviewed literature and governmental or non-governmental international regulatory body publications on LF in Zambia through a multiphase screening and data extraction process. Specifically, we aim to answer one primary question and several related secondary questions.

I. What are the predominant types of research on LF in Zambia that have been conducted?

- What organizations fund LF research in Zambia?
- What journals publish research on LF in Zambia?
- What are the seminal papers, as assessed by citation index, on LF in Zambia?

## Methods

A scoping review protocol was developed following PRISMA guidelines for Scoping Reviews [16] (Appendix 1) and using JBI Scoping Review Methodology Group’s guidance on conducting scoping reviews [17, 18].

### Inclusion and exclusion criteria

The term ‘articles’ here will be used to mean peer-reviewed literature published in journals and government or international regulatory body publications (e.g. WHO). Inclusion criteria were defined through primary and secondary criteria. We will not exclude papers based on publication date.

Inclusion criteria:

I. Primary criteria

- Articles in English or translated to English.

II. Secondary criteria

- Articles with a core or significant focus on LF

   (i) Expanded during title-abstract screen to include broader categories LF is nested within (e.g., neglected tropical diseases, filariae, etc.)

ii. Articles with a core or significant focus on the Zambian context.

   (i) Expanded during title-abstract screen to include broader categories Zambia is nested within (e.g., Sub-Saharan Africa, Southern Africa, etc.)

Exclusion criteria:

I. Articles with a focus on Zambia that do not include a thorough discussion of LF.

   i. ‘Thorough’ operationalized to mean ‘contains LF-related discussion or data, including incidence, prevalence, GIS data, etc.’

II. Articles with a focus on LF that do not include a thorough discussion of the Zambian context.

   i. ‘Thorough’ operationalized to mean ‘contains discussion or data that is specific to Zambia or includes Zambia among a set of data or discussions of multiple countries/contexts’.

III. Articles that are not written in or translated to English.
IV. Texts that are not published in peer-reviewed journals or by government or international regulatory bodies.
V. Texts published primarily as conference proceedings or poster abstracts.
VI. Book chapters
VII. Articles with full text unavailable, inaccessible, or unlocatable.

### Search strategy

Records will be gathered through two primary strategies: database search and supplement non-journal article inclusion. The database search was conducted using PubMed, Embase, Web of Science, Cochrane CENTRAL, WHO ICTRP, Pan African Clinical Trials Registry, and ClinicalTrials.gov. The search terms used for each database are defined in Appendix 2 and the initial literature search was performed on 2/27/2023. Non-journal articles have not been gathered at this point.

### Record management

Records will be uploaded into Rayyan [19]. Duplicate records will be deleted prior to the title-abstract screening phase. Following the screening phase, article data will be stored in Microsoft Excel.

### Screening strategy

The screening process will consist of 2 phases, title-abstract screening and full-text screening. Prior to initiating the title-abstract screen, a test batch of 20 articles will be used to train screeners. Concordance between screeners greater than 90% was used to determine readiness for the initiation of the screening process. Two reviewers will independently review each record for inclusion or exclusion in both the title-abstract and full-text screening phases. Ties will be broken by a third reviewer.

In the title-abstract screening phase, the primary inclusion criterion must be met and one of the secondary inclusion criteria must be met. This is to ensure that articles that may include a thorough discussion of LF in Zambia in the body of the text but might not otherwise exclusively mention either keyword in the title or abstract will not be screened out based on their discussion of either LF or Zambia alone in the title or abstract. Additionally, broader categories that include LF or Zambia (e.g., neglected tropical diseases, Sub-Saharan Africa, etc.) were used during the title-abstract screen to ensure that papers that may not mention LF or Zambia explicitly but may otherwise include their thorough discussion in the main text, were not screened out. During the full-text screening phase, two screeners will review each full text. All inclusion criteria must be met and none of the exclusion criteria must be met to justify inclusion in the full-text analysis phase.

### Data extraction

The full-text data extraction will use a standardized format following a codebook adapted from another scoping review performed by this group, Grifferty et al. [20], which facilitates intercoder reliability and defines papers across study themes and types. Study themes describe the article’s core focus as they relate to LF, such as epidemiology or pathophysiology. Study type describes the methodology used in that article, such as systematic review or clinical trial. The general study themes and types are outlined in Table 1. Data will be imported into Microsoft Excel for analysis and summarization.

**Table 1:** Primary and secondary items used to categorize articles screened in following title-abstract and full text screening. Each peer reviewed article will undergo data extraction using this table as a guide. “Study theme” refers to the overall focus of article, while “study type” refers to the methodology. “Number of citations” will be acquired using estimates from Google scholar.

Primary Item	Secondary Items
Title	
Year of publication	
Journal / Conference / Meeting / Congress	
Database(s)	
Study theme	General epidemiology, prevention, pathophysiology, diagnostics, treatment, health systems/policy, vectors, co-infections, general topics
Study type	Basic science, clinical research, epidemiological research, secondary research, government, or institutional report
Number of citations	
Funding Source(s)	

### Data summarization and presentation

Summary statistics describing the spread of relevant literature across the study themes and types will be presented, along with summary statistics of the year of publication, number of citations, publishing bodies, and funding sources. The number of citations will be drawn from Google Scholar citation estimates. Recommendations on potential future directions will be guided by these summary statistics and by expert input from the authors and literature.

## Discussion

The scoping review will look at literature published at any time with the goal of capturing a broad initial repository of potentially relevant studies. The title-abstract screening phase is designed to ensure that articles that may contain relevant information will not be screened out, increasing the likelihood of yielding a more complete final set of texts. The manual addition of known relevant government and international regulatory body documents that may not otherwise appear in the queried databases will further improve the final yield. We are excluding book chapters, posters and conference abstracts which may result in the loss of some relevant information. Additionally, we will only include English texts, which will result in the loss of relevant literature published in other languages.

Study dissemination will be accomplished through submission of a manuscript to a relevant journal. Prior to submission, the study has been registered in OSF [21]. Amendments to the study protocol will be made openly available through the OSF platform.

LF is a neglected tropical disease that is endemic in many countries, including Zambia. Local governments and other key stakeholders, including GPELF, have made great strides in reducing the burden of LF in Zambia, especially over the last two decades. The scope of research on the disease in Zambia is not well understood. This scoping review is designed to fill this gap and provide crucial knowledge about our understanding of the disease in the Zambian context.

We anticipate that, through this scoping review, we will be able to capture most of the research pertaining to LF in Zambia and code that research according to study type and theme. We will describe our findings through various summary statistics and use them to define potential research gaps that can fuel future research direction. Now, in the NTD 2021–2030 era, evaluating our understanding of LF in Zambia will provide key insights into the epidemiology and programming of the disease in a country where interventions are ongoing.

## Supporting information

S1 Checklist Preferred Reporting Items for Systematic reviews and Meta-Analyses extension for Scoping Reviews (PRISMA-ScR) checklist. (DOCX)

S1 File Search strings and result summary. (DOCX)
