# Supplementary material for: Lymphatic filariasis in Zambia: A scoping review protocol
Source: PLoS One. 2023 Oct 4;18(10):e0292237. doi: 10.1371/journal.pone.0292237 (PMC10550120; doi:10.1371/journal.pone.0292237)
Supplement: S1 File — (DOCX) [file pone.0292237.s002.docx]

**PubMed**

("Elephantiasis, Filarial" [Mesh] OR "Lymphatic filariasis" OR "neglected tropical diseases" OR "Wuchereria bancrofti" OR "elephantiasis" OR "Lymphedema"[Mesh:NoExp] OR "lymphoedema" OR "lymphedema" OR "Testicular Hydrocele" [Mesh] OR "hydrocele" OR "nchofu" OR "nthumbo" OR "pholo") AND ("Zambia" [Mesh] OR "Zambia" OR "Zambian" OR "Africa South of the Sahara" [Mesh] OR "sub-Saharan Africa" OR "Southern Africa" OR "Africa")

Results: 2265

Date searched: 2/27/23

Filters: None

**Embase**

('Lymphatic filariasis'/exp OR 'Lymphatic filariasis' OR 'neglected tropical diseases' OR 'Wuchereria bancrofti'/exp OR 'Wuchereria bancrofti' OR 'elephantiasis' OR 'lymphedema'/exp OR 'lymphoedema' OR 'lymphedema' OR 'hydrocele'/exp OR 'hydrocele' OR 'nchofu' OR 'nthumbo' OR 'pholo') AND ('Zambia'/exp OR 'Zambia' OR 'Zambian' OR 'Africa south of the Sahara'/exp OR 'sub-Saharan Africa' OR 'Southern Africa' OR 'Africa') AND ('article'/it OR 'article in press'/it OR 'preprint'/it OR 'review'/it)

Results: 4203

Date searched: 2/27/23

Filters: None

**Web of Science**

("Lymphatic filariasis" OR "neglected tropical diseases" OR "Wuchereria bancrofti" OR "elephantiasis" OR "lymphedema" OR "lymphoedema" OR "hydrocele" OR "nchofu" OR "nthumbo" OR "pholo") AND ("Zambia" OR "Zambian" OR "Africa South of the Sahara" OR "sub-Saharan Africa" OR "Southern Africa" OR "Africa")

Results: 4376

Date searched: 2/27/23

Filters: Document Type: Exclude: Meeting, Editorial Material, Letter, Biography, Books, News, Patent

**Cochrane CENTRAL**

#1 MeSH descriptor: [Elephantiasis, Filarial] explode all trees

#2 MeSH descriptor: [Lymphedema] this term only

#3 MeSH descriptor: [Testicular Hydrocele] explode all trees

#4 "Lymphatic filariasis" OR "neglected tropical diseases" OR "Wuchereria bancrofti" OR "elephantiasis" OR "lymphoedema" OR "lymphedema" OR "hydrocele" OR "nchofu" OR "nthumbo" OR "pholo"

#5 MeSH descriptor: [Zambia] explode all trees

#6 MeSH descriptor: [Africa South of the Sahara] explode all trees

#7 "Zambia" OR "Zambian" OR "sub-Saharan Africa" OR "Southern Africa" OR "Africa"

#8 #1 OR #2 OR #3 OR #4

#9 #5 OR #6 OR #7

#10 #8 AND #9

Results: 191

Date searched: 2/27/23

Filters: None

**WHO’s Global Index Medicus**

("Lymphatic filariasis" OR "neglected tropical diseases" OR "Wuchereria bancrofti" OR "elephantiasis" OR "lymphedema" OR "lymphoedema" OR "hydrocele" OR "nchofu" OR "nthumbo" OR "pholo") AND ("Zambia" OR "Zambian" OR "Africa South of the Sahara" OR "sub-Saharan Africa" OR "Southern Africa" OR "Africa")

Results: 35

Date searched: 2/27/23

Filters: None

**WHO’s ICTRP**

Lymphatic filariasis = Condition

Recruiting country = Zambia

Recruitment status = All

Results: 0

Date searched: 2/27/23

**ClinicalTrials.gov**

Condition or disease = lymphatic filariasis

Locations - Country = Zambia

Results: 0

Date searched: 2/27/23

**Total results:**

11,070 prior to deduplication

6,363 after deduplication
